# Genome sequence of the moderately thermophilic halophile *Flexistipes sinusarabici* strain (MAS10^T^)

**DOI:** 10.4056/sigs.2235024

**Published:** 2011-09-23

**Authors:** Alla Lapidus, Olga Chertkov, Matt Nolan, Susan Lucas, Nancy Hammon, Shweta Deshpande, Jan-Fang Cheng, Roxanne Tapia, Cliff Han, Lynne Goodwin, Sam Pitluck, Konstantinos Liolios, Ioanna Pagani, Natalia Ivanova, Marcel Huntemann, Konstantinos Mavromatis, Natalia Mikhailova, Amrita Pati, Amy Chen, Krishna Palaniappan, Miriam Land, Loren Hauser, Evelyne-Marie Brambilla, Manfred Rohde, Birte Abt, Stefan Spring, Markus Göker, James Bristow, Jonathan A. Eisen, Victor Markowitz, Philip Hugenholtz, Nikos C. Kyrpides, Hans-Peter Klenk, Tanja Woyke

**Affiliations:** 1 DOE Joint Genome Institute, Walnut Creek, California, USA; 2 Los Alamos National Laboratory, Bioscience Division, Los Alamos, New Mexico, USA; 3 Biological Data Management and Technology Center, Lawrence Berkeley National Laboratory, Berkeley, California, USA; 4 Oak Ridge National Laboratory, Oak Ridge, Tennessee, USA; 5 DSMZ - German Collection of Microorganisms and Cell Cultures GmbH, Braunschweig, Germany; 6 HZI – Helmholtz Centre for Infection Research, Braunschweig, Germany; 7 University of California Davis Genome Center, Davis, California, USA; 8 Australian Centre for Ecogenomics, School of Chemistry and Molecular Biosciences, The University of Queensland, Brisbane, Australia

**Keywords:** strictly anaerobic, Gram-negative, non-motile, heterotrophic, moderately thermophilic, marine, brine, *Deferribacteraceae*, GEBA

## Abstract

*Flexistipes sinusarabici* Fiala *et al*. 2000 is the type species of the genus *Flexistipes* in the family *Deferribacteraceae*. The species is of interest because of its isolated phylogenetic location in a genomically under-characterized region of the tree of life, and  because of its origin from a multiply extreme environment; the Atlantis Deep brines of the Red Sea, where it had to struggle with high temperatures, high salinity, and a high concentrations of heavy metals. This is the fourth completed genome sequence to be published of a type strain of the family *Deferribacteraceae*. The 2,526,590 bp long genome with its 2,346 protein-coding and 53 RNA genes is a part of the *** G****enomic* *** E****ncyclopedia of* *** B****acteria and* *** A****rchaea * project.

## Introduction

Strain MAS10^T^ (= DSM 4947 = ATCC 49648) is the type strain of *Flexistipes sinusarabici* [1,2] which is the type and only species of the genus *Flexistipes* [1,2]. The strain was first isolated from the Atlantis II Deep brines of the Red Sea [1], together with four related isolates. The generic name derives from the Latin words *flexus*, a bending, turning, winding, and *stipes,* a branch of tree, stick [1]. The species epithet is derived from the Latin words *sinus,* a curve or fold in land, a gulf, and *arabicus*, Arabic, describing the place of isolation [1]. Since the time of its isolation in the late 1980s until now no closely related bacterium (16S rRNA identity >90%) was described. The resistance of the strain to moderate heat, high salt concentrations, and heavy metals [1] should make it an interesting target for extremophile biotechnology. Here we present a summary classification and a set of features for *F. sinusarabici* MAS10^T^, together with the description of the complete genomic sequencing and annotation.

## Classification and features

A representative genomic 16S rRNA sequence of strain MAS10^T^ was compared using NCBI BLAST [3,4] under default settings (e.g., considering only the high-scoring segment pairs (HSPs) from the best 250 hits) with the most recent release of the Greengenes database [5] and the relative frequencies of taxa and keywords (reduced to their stem [6]) were determined, weighted by BLAST scores. The most frequently occurring genera were *Acidithiobacillus* (60.0%), *Deferribacter* (26.8%), *Flexistipes* (8.2%), *Desulfuromonas* (2.2%) and *Calditerrivibrio* (1.8%) (80 hits in total). Regarding the single hit to sequences from members of the species, the average identity within HSPs was 98.0%, whereas the average coverage by HSPs was 96.9%. Among all other species, the one yielding the highest score was *Deferribacter abyssi* (AJ515881), which corresponded to an identity of 89.7% and an HSP coverage of 86.4%. (Note that the Greengenes database uses the INSDC (= EMBL/NCBI/DDBJ) annotation, which is not an authoritative source for nomenclature or classification.) The highest-scoring environmental sequence was FR744611 ('succession potential reducers nitrate-treated facility determined temperature and nitrate availability production water Halfdan oil field clone PWB039'), which showed an identity of 96.7% and an HSP coverage of 93.1%. The most frequently occurring keywords within the labels of all environmental samples which yielded hits were 'microbi' (3.9%), 'acid' (3.4%), 'sediment' (3.3%), 'water' (3.0%) and 'oil' (2.4%) (170 hits in total). The most frequently occurring keyword within the labels of those environmental samples which yielded hits of a higher score than the highest scoring species was 'avail, determin, facil, field, halfdan, nitrat, nitrate-tr, oil, potenti, product, reduc, success, temperatur, water' (7.1%) (1 hit in total). While these keywords fit to the marine environment from which strain MAS10^T^ originated, they also point to sediments and oil fields which were so far not considered as habitats for *F. sinusarabici*.

**Figure 1:**
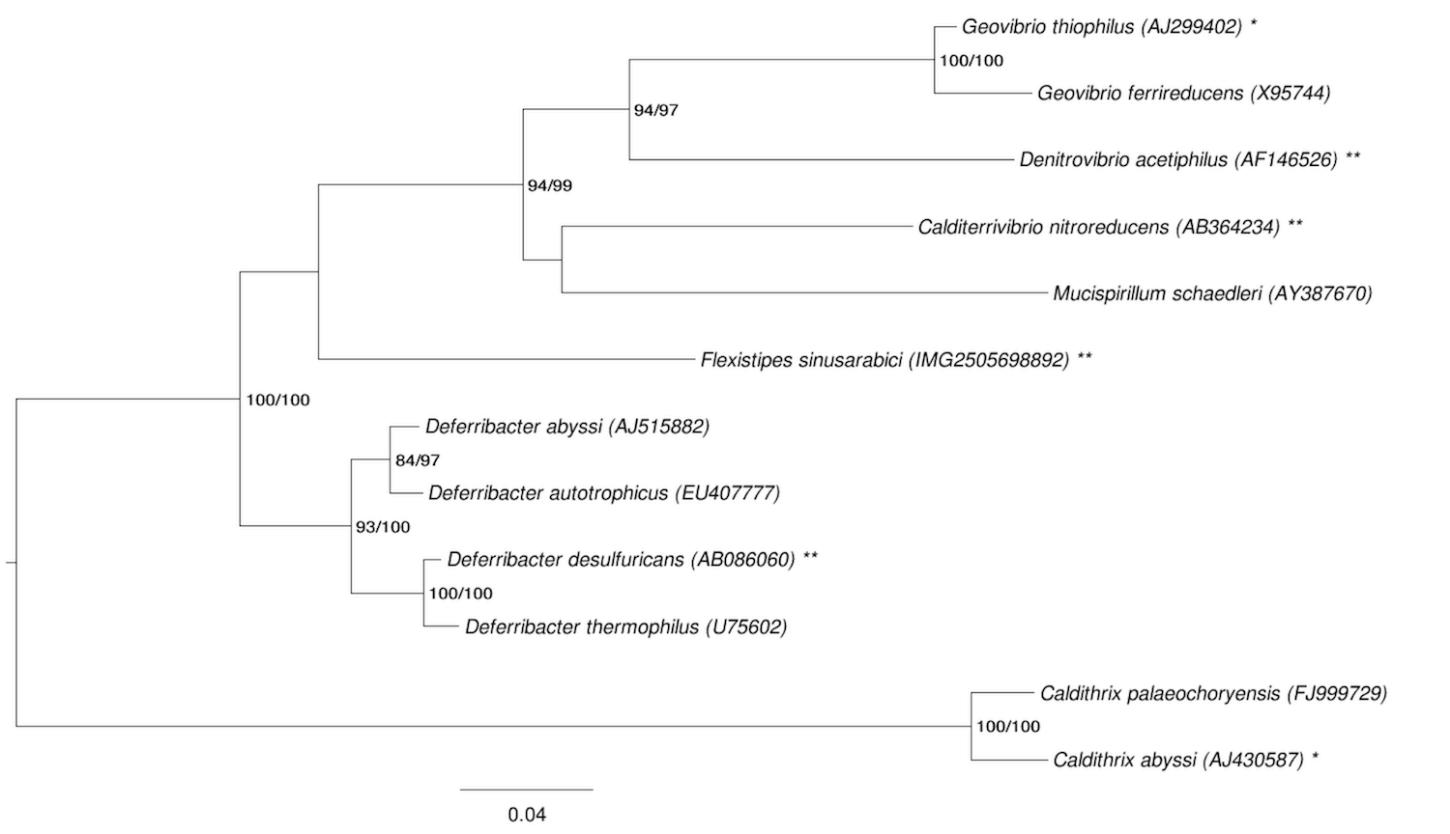
Phylogenetic tree highlighting the position of *F. sinusarabici* relative to the type strains of the other species within the phylum "*Deferribacteres*". The tree was inferred from 1,459 aligned characters [7,8] of the 16S rRNA gene sequence under the maximum likelihood (ML) criterion [9]. Rooting was done initially using the midpoint method [10] and then checked for its agreement with the current classification (Table 1). The branches are scaled in terms of the expected number of substitutions per site. Numbers adjacent to the branches are support values from 250 ML bootstrap replicates [11] (left) and from 1,000 maximum parsimony bootstrap replicates [12] (right) if larger than 60%. Lineages with type strain genome sequencing projects registered in GOLD [13] are labeled with one asterisk, those also listed as 'Complete and Published' with two asterisks [14-16].

Figure 1 shows the phylogenetic neighborhood of *F. sinusarabici* MAS10^T^ in a 16S rRNA based tree. The sequences of the two identical 16S rRNA gene copies in the genome differ by two nucleotides from the previously published 16S rRNA sequence M59231, which contains 25 ambiguous base calls.

**Table 1:** Classification and general features of *F. sinusarabici* MAS10^T^ according to the MIGS recommendations [17] and the NamesforLife database [18].

**MIGS ID**	**Property**	**Term**	**Evidence code**
	Current classification	Domain *Bacteria*	TAS [19]
		Phylum “*Deferribacteres*”	TAS [20,21]
		Class “*Deferribacteres*”	TAS [22,23]
		Order *Deferribacterales*	TAS [22,24]
		Family *Deferribacteraceae*	TAS [22,25]
		Genus *Flexistipes*	TAS [1,2]
		Species *Flexistipes sinusarabici*	TAS [1,2]
		Type strain MAS10	TAS [1]
	Gram stain	negative	TAS [1]
	Cell shape	straight to acutely bent rods	TAS [1]
	Motility	non-motile	TAS [1]
	Sporulation	none	TAS [1]
	Temperature range	30–53°C, moderately thermophilic	TAS [1]
	Optimum temperature	45–50°C	TAS [1]
	Salinity	at least 3% NaCl, growths with up to 18% NaCl	TAS [1]
MIGS-22	Oxygen requirement	strictly anaerobic	TAS [1]
	Carbon source	complex organic components like yeast extract, meat extract, peptone, tryptone	TAS [1]
	Energy metabolism	heterotrophic	TAS [1]
MIGS-6	Habitat	marine, deep brine water	TAS [1]
MIGS-15	Biotic relationship	free-living	TAS [1]
MIGS-14	Pathogenicity	none	TAS [1]
	Biosafety level	1	TAS [26]
	Isolation	interface between upper brine layer and deep sea water	TAS [1]
MIGS-4	Geographic location	Atlantis II Deep brines, Red Sea	TAS [1]
MIGS-5	Sample collection time	1987 or before	NAS
MIGS-4.1	Latitude	21.37	TAS [1]
MIGS-4.2	Longitude	38.07	TAS [1]
MIGS-4.3	Depth	2,000 – 2,200 m	TAS [1]
MIGS-4.4	Altitude	-2,200 – 2,200 m	TAS [1]

Evidence codes - TAS: Traceable Author Statement (i.e., a direct report exists in the literature); NAS: Non-traceable Author Statement (i.e., not directly observed for the living, isolated sample, but based on a generally accepted property for the species, or anecdotal evidence). These evidence codes are from the Gene Ontology project [27].

**Figure 2:**
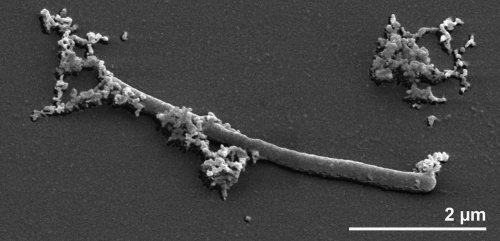
Scanning electron micrograph of *F. sinusarabici* MAS10^T^

Cells of strain MAS10^T^ are straight to bent rods, about 0.3 μm wide and 4–50 μm long (Figure 2) [1]. *F. sinusarabici* was described as non-motile [1]. Spore-formation was not observed [1]. MAS10^T^ cells stain Gram-negative, and growth is strictly anaerobic, with the best growth occurring within a temperature range of 45–50°C and a minimum doubling time of 8 ½ hours [1]. Optimal pH range for the strain is pH 6-8 [1]. Strain MAS10^T^ requires at least 3% NaCl for growth, but also grows at salt concentrations as high as 10% [1]. The organism prefers complex growth substrates such as yeast extract, meat extract, peptone and tryptone, while formate, lactate, citrate, malate, carbohydrate, amino acids and alcohols do not support cell growth [1]. Strain MAS10^T^ shows an unusual resistance against the transcription inhibitor rifampicin [1], which is however also commonly found among the spirochetes.

### Chemotaxonomy

The chemotaxonomic data for MAS10^T ^ is relatively sparse: No information on cell wall structure, quinones or polar lipids is available. The fatty acid composition is dominated by saturated unbranched acids: C_18_ (23.3%), C_16_ (15.1%), C_17_ (12.6%), with some branched acids *iso*-C_14_ (10.2%), *anteiso*-C_15_ (10.2%), *iso*-C_16_ (4.1%), *iso*-C_15_ (3.6%), and few unsaturated acids C_18:1_ (9.9%), C_16:1_ (2.8%), C_17:1_ (2.5%) [1].

## Genome sequencing and annotation

### Genome project history

This organism was selected for sequencing on the basis of its phylogenetic position [28], and is part of the *** G****enomic* *** E****ncyclopedia of* *** B****acteria and* *** A****rchaea * project [29]. The genome project is deposited in the Genome On Line Database [13] and the complete genome sequence is deposited in GenBank. Sequencing, finishing and annotation were performed by the DOE Joint Genome Institute (JGI). A summary of the project information is shown in Table 2.

**Table 2:** Genome sequencing project information

**MIGS ID**	**Property**	**Term**
MIGS-31	Finishing quality	Finished
MIGS-28	Libraries used	Four genomic libraries: one 454 pyrosequence standard library, two 454 PE libraries (3 kb, 15.5 kb insert size), one Illumina library
MIGS-29	Sequencing platforms	Illumina GAii, 454 GS FLX Titanium
MIGS-31.2	Sequencing coverage	162.0 × Illumina; 37.9 × pyrosequence
MIGS-30	Assemblers	Newbler version 2.3, Velvet version 0.7.63, phrap SPS-4.24
MIGS-32	Gene calling method	Prodigal 1.4, GenePRIMP
	INSDC ID	CP002858
	Genbank Date of Release	June 17, 2011
	GOLD ID	Gc01819
	NCBI project ID	45817
	Database: IMG-GEBA	2505679008
MIGS-13	Source material identifier	DSM 4947
	Project relevance	Tree of Life, GEBA

### Growth conditions and DNA isolation

*F. sinusarabici* MAS10^T^, DSM 4947, was grown anaerobically in DSMZ medium 524 (*Flexistipes* Medium) [30] at 47°C. DNA was isolated from 0.5-1 g of cell paste using Jetflex Genomic DNA Purification Kit (GENOMED 600100) following the standard protocol as recommended by the manufacturer, but adding 10µl proteinase K for one hour extended lysis at 58°C. DNA is available through the DNA Bank Network [31].

### Genome sequencing and assembly

The genome was sequenced using a combination of Illumina and 454 sequencing platforms. All general aspects of library construction and sequencing can be found at the JGI website [32]. Pyrosequencing reads were assembled using the Newbler assembler (Roche). The initial Newbler assembly consisting of 175 contigs in two scaffolds was converted into a phrap [33] assembly by making fake reads from the consensus, to collect the read pairs in the 454 paired end library. Illumina GAii sequencing data (489.7 Mb) was assembled with Velvet [34] and the consensus sequences were shredded into 2.0 kb overlapped fake reads and assembled together with the 454 data. The 454 draft assembly was based on 170.4 Mb 454 draft data and all of the 454 paired end data. Newbler parameters are -consed -a 50 -l 350 -g -m -ml 20. The Phred/Phrap/Consed software package [33] was used for sequence assembly and quality assessment in the subsequent finishing process. After the shotgun stage, reads were assembled with parallel phrap (High Performance Software, LLC). Possible mis-assemblies were corrected with gapResolution [32], Dupfinisher [35], or sequencing cloned bridging PCR fragments with subcloning. Gaps between contigs were closed by editing in Consed, by PCR and by Bubble PCR primer walks (J.-F. Chang, unpublished). A total of 605 additional reactions and 15 shatter libraries were necessary to close gaps and to raise the quality of the finished sequence. Illumina reads were also used to correct potential base errors and increase consensus quality using a software Polisher developed at JGI [36]. The error rate of the completed genome sequence is less than 1 in 100,000. Together, the combination of the Illumina and 454 sequencing platforms provided 199.9 × coverage of the genome. The final assembly contained 248,918 pyrosequence and 395,536,860 Illumina reads.

### Genome annotation

Genes were identified using Prodigal [37] as part of the Oak Ridge National Laboratory genome annotation pipeline, followed by a round of manual curation using the JGI GenePRIMP pipeline [38]. The predicted CDSs were translated and used to search the National Center for Biotechnology Information (NCBI) non-redundant database, UniProt, TIGR-Fam, Pfam, PRIAM, KEGG, COG, and InterPro databases. Additional gene prediction analysis and functional annotation was performed within the Integrated Microbial Genomes - Expert Review (IMG-ER) platform [39].

## Genome properties

**Figure 3:**
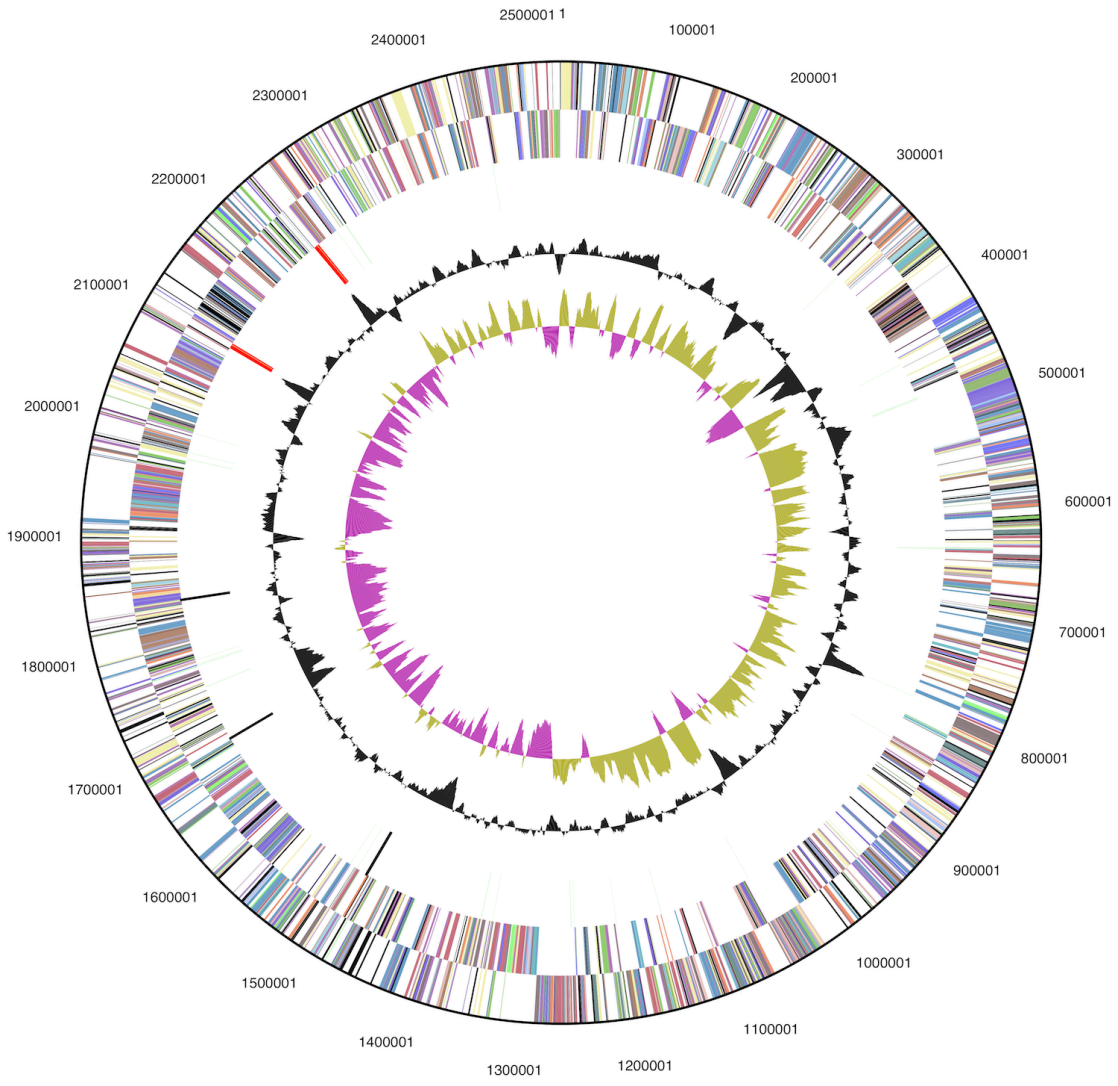
Graphical circular map of the chromosome. From outside to the center: Genes on forward strand (color by COG categories), Genes on reverse strand (color by COG categories), RNA genes (tRNAs green, rRNAs red, other RNAs black), GC content, GC skew.

The genome consists of a 2,526,590 bp long circular chromosome with a G+C content of 38.3% (Table 3 and Figure 3). Of the 2,399 genes predicted, 2,346 were protein-coding genes, and 53 RNAs; 85 pseudogenes were also identified. The majority of the protein-coding genes (75.2%) were assigned a putative function while the remaining ones were annotated as hypothetical proteins. The distribution of genes into COGs functional categories is presented in Table 4.

**Table 3:** Genome Statistics

**Attribute**	Value	% of Total
Genome size (bp)	2,526,590	100.00%
DNA coding region (bp)	2,179,830	86.28%
DNA G+C content (bp)	967,539	38.29%
Number of replicons	1	
Extrachromosomal Elements	0	
Total genes	2,399	100.00%
RNA genes	53	2.21%
rRNA operons	2	
Protein-coding genes	2,346	97.79%
Pseudo genes	85	3.54%
Genes with function prediction	1,803	75.16%
Genes in paralog clusters	242	10.09%
Genes assigned to COGs	1,924	80.20%
Genes assigned Pfam domains	1,978	82.45%
Genes with signal peptides	366	15.26%
Genes with transmembrane helices	579	24.14%
CRISPR repeats	0	

**Table 4:** Number of genes associated with the general COG functional categories

**Code**	**value**	**%age**	**Description**
J	145	7.0	Translation, ribosomal structure and biogenesis
A	1	0.1	RNA processing and modification
K	84	4.0	Transcription
L	205	9.8	Replication, recombination and repair
B	2	0.1	Chromatin structure and dynamics
D	21	1.0	Cell cycle control, cell division, chromosome partitioning
Y	0	0.0	Nuclear structure
V	26	1.3	Defense mechanisms
T	115	5.5	Signal transduction mechanisms
M	135	6.5	Cell wall/membrane/envelope biogenesis
N	36	1.7	Cell motility
Z	0	0.0	Cytoskeleton
W	0	0.0	Extracellular structures
U	62	3.0	Intracellular trafficking, secretion, and vesicular transport
O	81	3.9	Posttranslational modification, protein turnover, chaperones
C	174	8.3	Energy production and conversion
G	66	3.2	Carbohydrate transport and metabolism
E	198	9.5	Amino acid transport and metabolism
F	52	2.5	Nucleotide transport and metabolism
H	115	5.5	Coenzyme transport and metabolism
I	60	2.9	Lipid transport and metabolism
P	86	4.1	Inorganic ion transport and metabolism
Q	32	1.5	Secondary metabolites biosynthesis, transport and catabolism
R	246	11.8	General function prediction only
S	145	7.0	Function unknown
-	475	19.8	Not in COGs

## Insight into the genome sequence

### Comparative genomics

Lacking an available genome sequence of *Deferribacter abyssi*, the species yielding the highest score, the following comparative analyses were done with *D. desulfuricans* [14] (GenBank AP011529, AP011530) and *Calditerrivibrio nitroreducens* (GenBank CP002347, CP002348) [16], the phylogenetically closest organisms for which a genome sequence was available. The genomes of *F. sinusarabici*, *D. desulfuricans* and *C. nitroreducens* are similar in sizes (2.5 Mb, 2.5 Mb and 2.2 Mb, respectively) and have a similar, quite low G+C content (38%, 30% and 36%, respectively). Whereas *F. sinusarabici* has no plasmid, *D. desulfuricans* harbors a 5.9 kb plasmid; *C. nitroreducens* contains a 30.8 kb megaplasmid.

An estimate of the overall similarity between the three genomes was generated with the GGDC-Genome-to-Genome Distance Calculator [40,41]. This system calculates the distances by comparing the genomes to obtain HSPs (high-scoring segment pairs) and inferring distances from a set of formulas (1, HSP length / total length; 2, identities / HSP length; 3, identities / total length). Table 5 shows the results of the pairwise comparison between the three genomes.

**Table 5:** Pairwise comparison of *F. sinusarabici*, *D. desulfuricans* and *C. nitroreducens* using the GGDC-Calculator.

		1, HSP length / total length [%]	2, identities / HSP length [%]	3, identities / total length [%]
*F. sinusarabici*	*D. desulfuricans*	5.9	83.2	4.9
*F. sinusarabici*	*C. nitroreducens*	5.1	83.3	4.3
*D. desulfuricans*	*C. nitroreducens*	9.9	83.3	8.3

The comparison of the *F. sinusarabici* and *D. desulfuricans* genomes revealed that 5.9% of the average of both genome lengths are covered with HSPs. The identity within these HSPs was 83.2%, whereas the identity over the whole genome was only 4.9%. Similar results were inferred for *F. sinusarabici* and *C. nitroreducens* (Table 5). The genomes of *D. desulfuricans* and *C. nitroreducens* show a significantly higher degree of similarity with 9.9% of the average of both genomes are covered with HSPs of 83.3% identity. The identity over the whole length of the genomes was 8.3%. These values corroborate the relationship between the three organisms as shown in the 16S rRNA-based phylogenetic tree in Figure 1, as there is no bootstrap support that *F. sinusarabici* is closer related to either *C. nitroreducens* or *D. desulfuricans*.

**Figure 4:**
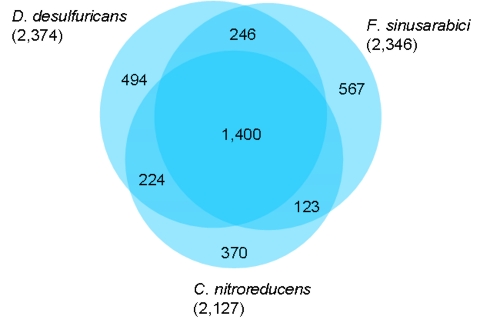
Venn diagram depicting the intersections of protein sets (total number of derived protein sequences in parentheses) of *F. sinusarabici, D. desulfuricans* and *C. nitroreducens*.

The fraction of shared genes in the three genomes is shown in a Venn diagram (Figure 4). The numbers of pairwise shared genes were calculated with the phylogenetic profiler function of the IMG/ER platform [33]. The homologous genes within the genomes were detected with a maximum E-value of 10^-5^ and a minimum identity of 30%. Roughly 61% of all genes in the genomes (1,400 genes) are shared by all three genomes, with about equal numbers of genes (224 and 246) shared on a pairwise basis by *F. sinusarabici* and *D. desulfuricans* or by *D. desulfuricans* and *C. nitroreducens*, respectively, and to the exclusion of the third genome. Within the 567 unique genes of *F. sinusarabici* that have no detectable homologs in the genomes of *D. desulfuricans and C. nitroreducens* (under the sequence similarity thresholds used for the comparison) the 86 genes (3.7% based on the whole gene number) encoding transposases appear to be noteworthy.

A remarkable difference between the compared organisms is their motility. Whereas *F. sinusarabici* is described to be non-motile, *D. desulfuricans* is motile by twitching [14] and *C. nitroreducens* is also described to be motile [16]. The mechanism of twitching motility is still unknown but it is thought that moving across surfaces is caused by extension and retraction of type IV pili. A set of genes that is responsible for twitching motility was identified in several organisms; in *Pseudomonas aeruginosa* a gene cluster involved in pilus biosynthesis and twitching motility was characterized, the gene products of this gene cluster show a high degree of sequence similarity to the chemotaxis (che) proteins of enterics and the gliding bacterium *Myxococcus xanthus* [42]. A closer look into the genome sequences of *F. sinusarabici, D. desulfuricans* and *C. nitroreducens* revealed the presence of different gene sets coding for chemotaxis proteins. In contrast to *D. desulfuricans* and *C. nitroreducens*, *F. sinusarabici* lacks four *che* genes (*che*B, *che*R, *che*V, *che*W). In *P. aeruginosa* a mutation in the *pil*I gene, a homolog to *che*W, lead to a blocking of pilus production [42]. It can be assumed that the missing *che*W gene in *F. sinusarabici* might be responsible for the non-motility of the cells, despite the rather large number of 36 genes annotated in the cell motility category of table 4.
